# Case 2/2016 - 76-Year-Old Male with Hypertensive Heart Disease, Renal Tumor and Shock

**DOI:** 10.5935/abc.20160067

**Published:** 2016-05

**Authors:** Marcela Anhesini Benetti, Rafael Amorim Belo Nunes, Luiz Alberto Benvenuti

**Affiliations:** Instituto do Coração (InCor) HC-FMUSP, São Paulo, SP - Brazil

**Keywords:** Hypertension, Cardiomyopathies, Defibrillators, Implantable, Kidney Neoplasms

The patient is a 76-year-old male with heart disease, hospitalized due to shock and respiratory failure.

At the age of 50 years, he was referred to InCor to investigate non-anginal chest pain. His exercise test was negative for ischemia.

Physical examination at that time was normal, except for slightly elevated blood pressure (BP: 140/90 mm Hg) and obesity (weight of 95 kg, height of 1.70 m; body mass index = 32.9kg/m^2^).

Electrocardiogram revealed sinus bradycardia, and the new exercise test was negative.

Echocardiogram on December 5, 1985, revealed normal valves and the following parameters: aortic root, 35 mm; left atrium, 44 mm; right ventricle, 21 mm; left ventricle, 53 mm; left ventricular ejection fraction, 60%; septal and posterior wall thickness, 11 mm.

Laboratory tests on December 6, 1985, were as follows: hemoglobin, 13.1 g/dL; hematocrit, 41%; leukocytes, 7,100/mm^3^; platelets, 268,000/mm^3^; glycemia, 118 mg/dL; creatinine, 1 mg/dL; sodium, 140 mEq/L; potassium, 4.5 mEq/L; total bilirubin, 0.54 mg/dL; direct bilirubin, 0.15 mg/dL; ALT, 76 IU/L; alkaline phosphatase, 227 IU/L (normal< 170 IU/L); total proteins, 78 g/dL; albumin, 4.3 g/dL; and globulins, 3.5 g/dL.

The patient was lost to follow-up at Incor until March 1996, when, at the age of 60 years, he experienced chest pressure, sweating and dyspnea on exertion, which, in a few weeks, progressed to dyspnea at rest. On that occasion, he reported smoking and having arterial hypertension, hyperuricemia, and lost his father due to myocardial infarction, and his mother due to stroke.

His physical examination on March 6, 1996, was normal, showing: weight, 104 kg; height, 1.70 m; BP, 160/90 mm Hg; heart rate, 80 bpm. On that same day, his ECG revealed sinus rhythm, heart rate of 78 bpm and right bundle-branch block (RBBB) (Figure 1), and his laboratory tests were as follows: urea, 20 mg/dL; creatinine, 1.3 mg/dL; and normal levels of myocardial injury markers.

**Figure 1:**
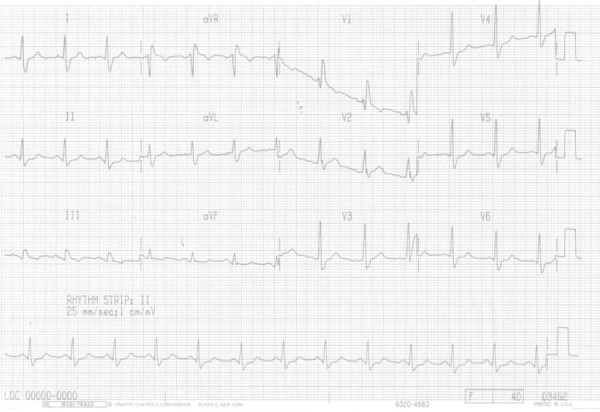
ECG: sinus rhythm, right bundle-branch block.

Exercise test on March 8, 1996, showed: maximum heart rate achieved, 139 bpm; duration of 5 min; initial BP, 150/90 mm Hg, and at peak exercise, 186/100 mm Hg, with no ischemic changes.

Echocardiogram on March 5, 1996, revealed left ventricular apical and laterobasal hypokinesia, and ejection fraction of 70%.

Coronary angiography on March 11, 1996, revealed 50% lesions in the anterior descending and circumflex branches of the left coronary artery. Ventriculography showed moderate diffuse hypokinesia.

Atenolol (50 mg), enalapril (10 mg) and acetylsalicylic acid (100 mg) were prescribed.

New exercise test on February 17, 1998, was negative for ischemia, and laboratory tests showed: cholesterol, 158 mg/dL; HDL-C, 24 mg/dL; LDL-C, 65 mg/dL; triglycerides, 347 mg/dL; glycemia, 105 mg/dL; uric acid, 7.4 mg/dL; creatinine, 1.0 mg/dL.

Echocardiogram on that same day revealed septal and posterior wall thickness of 12 mm, left ventricle of 52 mm, ejection fraction of 63%, and normal motility.

The patient remained asymptomatic from the cardiovascular viewpoint for more than 10 years until, at the age of 72 years, he experienced dyspnea that rapidly progressed in 20 days to dyspnea at rest, accompanied by tachycardic palpitations. He was admitted to another hospital, diagnosed with heart failure and tachycardia with wide QRS complex, initially identified as supraventricular with aberrancy, and then, as sustained ventricular tachycardia, which was reversed with amiodarone. He reported undergoing surgery to treat a malignant neoplasm of the urinary bladder at the age of 68 years. In addition, he reported undergoing gastrectomy because of peptic ulcer, but did not inform exactly when.

Electrocardiogram on August 7, 2008, showed tachycardia with wide QRS complex, heart rate of 150 bpm, with positive QRS from V_1_ to V_6_ (Figure 2). After reversion, the ECG evidenced RBBB with atrioventricular dissociation (Figure 3).

**Figure 2:**
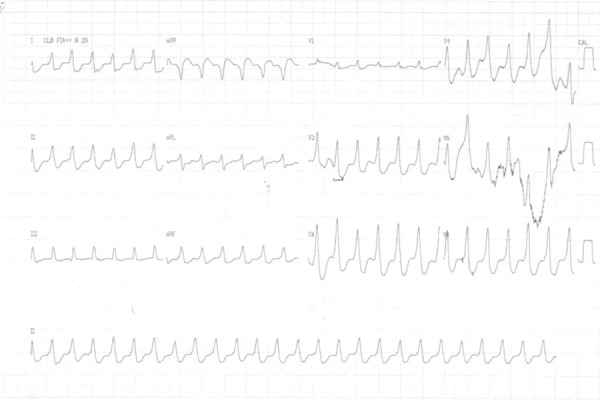
ECG: ventricular tachycardia and pure R waves from V1 to V6.

**Figure 3:**
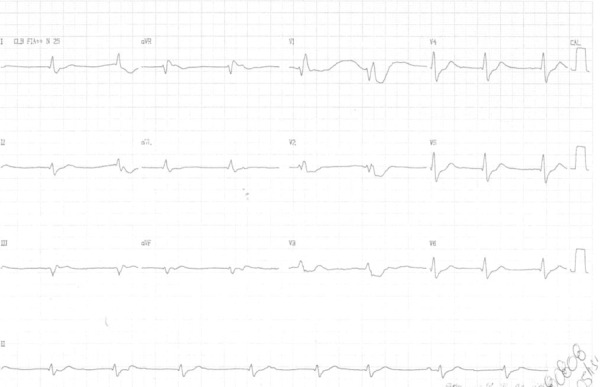
ECG: atrioventricular dissociation, right bundle-branch block.

Physical examination on September 5, 2008, revealed BP of 160/90 mm Hg, heart rate of 68 bpm, normal pulmonary and cardiac auscultations, and mild lower limb edema.

Electrocardiogram on September 5, 2008, revealed sinus rhythm, RBBB and multifocal ventricular extrasystoles (Figure 4).

**Figure 4:**
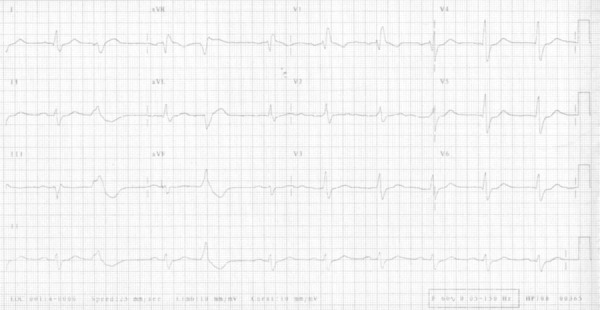
ECG: sinus rhythm, right bundle-branch block.

Carvedilol (6.25 mg), furosemide (40 mg), enalapril (20 mg) and amiodarone (200 mg) were prescribed to the patient, who was referred to the arrhythmia outpatient clinic.

Laboratory tests in February 2009 revealed: hemoglobin, 9.8 g/dL; hematocrit, 34; VCM, 65 fL; RDW, 20.3%; leukocytes, 8,000/mm^3^; platelets, 377,000/mm^3^; total cholesterol, 117 mg/dL; HDL-C, 25 mg/dL; LDL-C, 62 mg/dL; triglycerides, 123 mg/dL; glycemia, 125 mg/dL; creatinine, 1.72 mg/dL; urea, 48 mg/dL; sodium, 137 mEq/L; potassium, 5.3 mEq/L; calcium, 13 mg/dL; ionic calcium, 1.9 mmol/L; TSH, 2.4 *µ*U/mL; TP (INR), 1.0; TTPA (rel), 1.05; iron, 23 *µ*g/dL; transferrin saturation, 5%. Urinalysis: density, 1009; pH, 6.0; leukocytes, 2,000/mL; and red blood cells, 2,000/mL.

Coronary angiography on February 3, 2009, evidenced irregularities in the anterior descending branch of the left coronary artery, 60% in the first diagonal branch, and irregularities in the circumflex artery and right coronary artery. The left ventricle was dilated and had moderate diffuse hypokinesia. Hemodynamic parameters were: aorta (S/D/M) 160/80/107 mm Hg; left ventricle (S/D/ED) 160/05/20 mm Hg.

The patient was admitted in February 2009 for electrophysiological study, and was not on amiodarone at that time.

Upper digestive endoscopy on February 12, 2009, showed a previously operated on stomach (Billroth I gastrectomy) and severe alkaline reflux gastritis.

During electrophysiological study on February 13, 2009, extra-stimuli induced badly-tolerated monomorphic ventricular tachycardia [DI(+), aVL(+/-), lower axis, with positivity from V_1_ to V_6_, no transition] with degeneration to fibrillation after reversion attempt with burst. Successful defibrillation obtained with 200 J.

Echocardiogram on February 19, 2009, showed: diameters of the aorta and left atrium, 34 mm and 55 mm, respectively; septum, 12 mm; posterior wall, 10 mm; left ventricle diameters, 59/42 mm; left ventricular ejection fraction, 35%; akinesia of the apical and inferolateral walls; hypokinesia of the other walls; and severe mitral regurgitation.

Abdominal ultrasound on February 20, 2009, revealed dilation of the infrarenal aorta, and stone and nodule in the lower pole of the right kidney. Abdominal computed tomography on February 25, 2009, revealed a solid nodule in the lower pole of the right kidney, measuring 2.9x2.9 cm, with contrast uptake.

Implantable cardioverter defibrillator (ICD) was indicated as primary prophylaxis of sudden death and to support beta-blocker use, because the patient experienced bradycardia and arterial hypotension when an increase in beta-blocker dose was attempted.

The ICD implantation was performed on February 17, 2012. The patient was referred to a urologist and discharged from the hospital with the following daily prescription: carvedilol, 50 mg; hydralazine, 75 mg; hydrochlorothiazide, 25 mg; atorvastatin, 10 mg; amiodarone, 200 mg; isosorbide mononitrate, 20 mg; omeprazole, 20 mg; and ferrous sulfate, 80 mg.

During the patient's follow-up on an outpatient clinic basis, he developed dyspnea on moderate exertion.

On ICD assessment in March 2012, the device was functioning normally and had recorded one shock in February 2011 during an episode of ventricular tachycardia. The patient was hospitalized again on June 17, 2012, with consciousness lowering and arterial hypotension for one day.

On admission, the patient was drowsy, dehydrated (+/4+) and pale (2+/4+), and had non-productive cough, and neither fever nor dyspnea. His BP was 80/60 mm Hg, heart rate, 60 bpm, and room air O_2_ saturation ranging from 88% to 90%. Pulmonary auscultation revealed crepitant rales on the middle third of the right hemithorax. Cardiac auscultation showed irregular rhythm, low heart sounds and no heart murmur. The abdomen showed no change. There was no edema and the pulses were thin. Glasgow coma scale was as follows: eyes - opens eyes in response to voice (3); verbal - confused, disoriented (4); motor - obeys commands (6); no motor deficit; equal pupils reactive to light.

Volume administration and antibiotic therapy with ceftriaxone and clarithromycin were initiated.

Laboratory tests on June 17, 2012, revealed: hemoglobin, 11.1 g/dL; hematocrit, 36%; leukocytes, 10,040 (10% band neutrophils, 77% segmented neutrophils, 4% eosinophils, 8% lymphocytes, 1% monocytes); platelets, 110,000/mm^3^; PCR, 79.16 mg/L; CK-MB, 2.45 ng/mL; troponin I < 0.006 ng/mL; urea, 135 mg; creatinine, 4.55 mg/dL; sodium, 140 mEq/L; potassium, 3.3 mEq/L; calcium, 6.5 mEq/L; magnesium, 2.0 mEq/L; BNP, 273 pg/mL; total bilirubin, 0.54 mg/dL; direct bilirubin, 0.27 mg/dL; TP(INR) 1.1; TTPA(rel) 1.18.

Those antibiotics were replaced by the piperacillin-tazobactam association, and later, by vancomycin.

Laboratory tests on June 19, 2012, were as follows: hemoglobin, 10.9 g/dL; hematocrit, 34%; VCM, 89 fL; leukocytes, 7,050/mm^3^ (neutrophils 85%, eosinophils 2%, lymphocytes 9%, 4% monocytes); platelets, 79,000/mm^3^; PCR, 121.15 mg/dL; urea, 135 mg/dL; creatinine, 4.27 mg/dL (glomerular filtration: 14 mL/min/1.73m^2^); magnesium, 1.90 mEq/L; sodium, 137 mEq/L; potassium, 3.5 mEq/L; ionized calcium, 1.69 mmol/L; venous lactate, 13 mg/dL; venous pH, 7.33; venous bicarbonate, 23 mEq/L.

Despite treatment with volume administration, antibiotics and vasoactive amines, the patient remained shocked and died on June 20, 2012.

## Clinical aspects

The patient had multiple comorbidities, chest pain and dyspnea on exertion. During follow-up, cardiomyopathy installed with segmental impairment of left ventricular contractility and complex ventricular arrhythmia. Initially, he had arterial hypertension and sinus bradycardia, with no other significant changes. Later, the patient experienced clinical worsening and new comorbidities: hyperuricemia, smoking and obesity. The ECG showed a significant change over time, and RBBB appeared. The functional assessment of myocardial ischemia revealed no significant change during follow-up. The symptoms progressively worsened, and, at the age of 72 years, the patient was hospitalized due to sustained ventricular tachycardia with hemodynamic instability. He reported previous surgery to treat malignant urinary bladder neoplasm, and gastrectomy to treat peptic ulcer. In 2011, the patient received an ICD to prevent sudden death, the last assessment being in March 2012. In June 2012, he was hospitalized due to presumed septic shock and eventually died.

During follow-up, the patient developed progressive cardiomyopathy with segmental impairment of left ventricular contractility associated with complex ventricular arrhythmias, and no significant coronary artery disease.

The most likely cause of disease progression in the absence of coronary artery disease would be hypertensive heart disease,^1^ considering that the patient's age does not match the age group of cardiac impairment due to Chagas disease.^2,3^

Some other forms of cardiomyopathy are also characterized by segmental impairment of ventricular contractility and arrhythmogenic potential. Arrhythmogenic right ventricular cardiomyopathy/dysplasia is a genetically determined heart disease, in which myocytes change into adipose tissue and fibrosis, resulting in high risk for ventricular arrhythmias, sudden death and heart failure; however, it affects young individuals.^4,5^

Segmental impairment of ventricular contractility is also described in other forms of non-ischemic cardiomyopathy, such as dilated/idiopathic cardiomyopathy, being associated with an increased risk for arrhythmic events.^6^

In this patient, an ICD was implanted for primary prevention of sudden death due to arrhythmia.^7,8^ Although some studies have shown ICD implantation to reduce mortality in patients with history of sustained ventricular tachycardia, ventricular fibrillation or risk factors for sudden death, such as severe left ventricular dysfunction,^9,10^ the implantation of that device is associated with an increased risk for infections, which can result in high morbidity and mortality, especially when related to the device's infection.^11-16^ (**Marcela Anhesini Benetti, MD, and Rafael Amorim Belo Nunes, MD**)

## Diagnostic hypotheses

- Septic shock with undefined origin and non-responsive to the therapies used;

- Death due to infectious complications in an individual with non-ischemic cardiomyopathy and ICD, the presumed origin being the lungs or ICD-related. (**Marcela Anhesini Benetti, MD, and Rafael Amorim Belo Nunes, MD**)

## Postmortem examination

**Figure 5:**
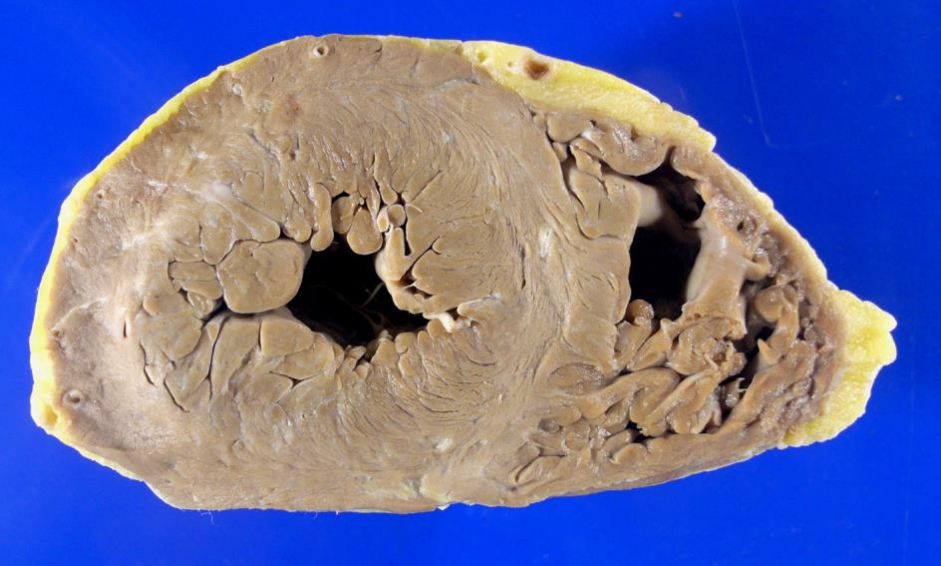
Cross-section of the heart at the ventricular level. There is mild left ventricular hypertrophy, with no cavitary dilation. Note the absence of areas of acute or healed myocardial infarction.

**Figure 6:**
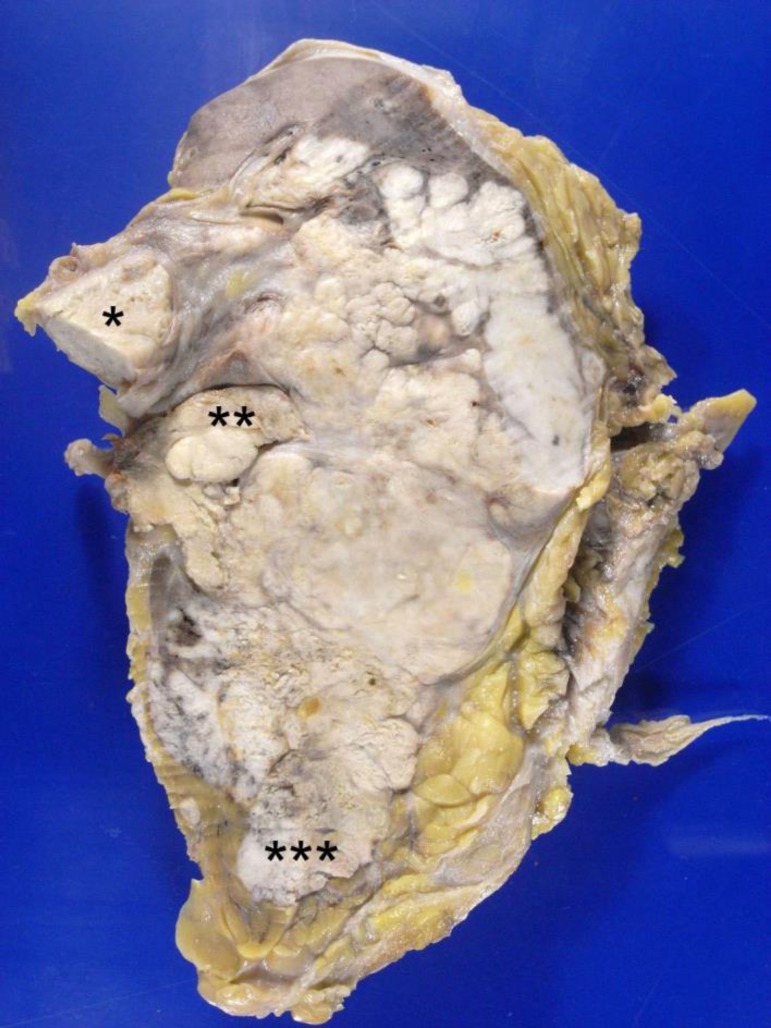
Right kidney section evidencing extensive whitish tumor infiltrating the parenchyma and invading the renal vein (asterisk), pelvis (double asterisk) and perirenal fat (triple asterisk).

**Figure 7:**
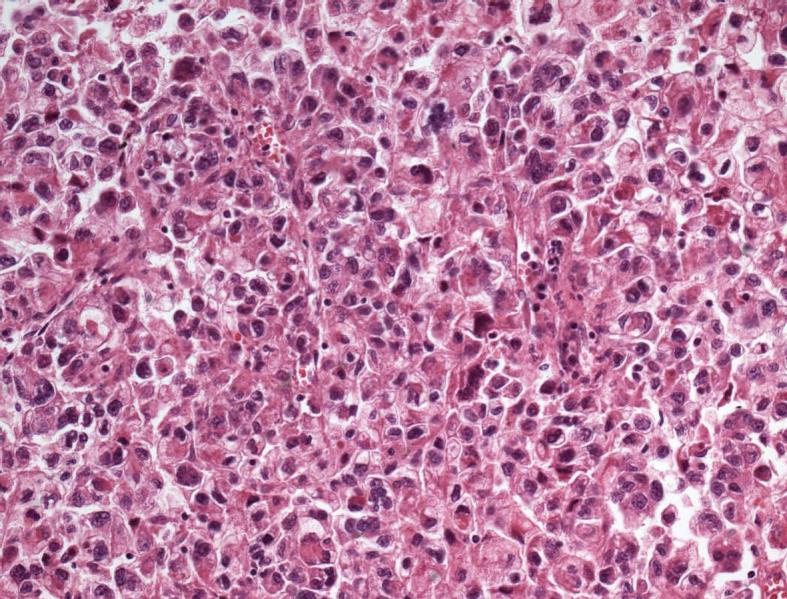
Microscopic section of the renal tumor. There is mild cell cohesion, intense anaplasia and mitotic figures. The histological aspect is compatible with renal cell carcinoma. Hematoxylin-Eosin, X 250.

**Figure 8:**
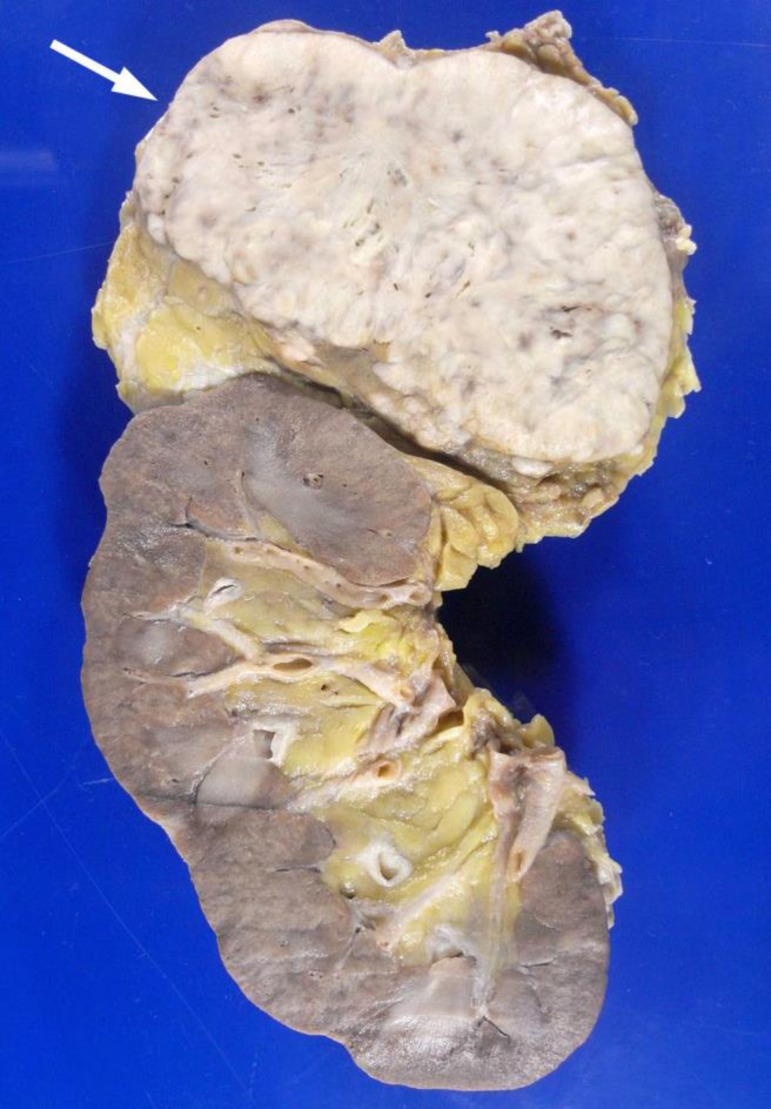
Left kidney section showing tumor close to the upper lobe (arrow) and complete replacement of the adrenal gland.

The heart weighed 558 g. The ICD's lead was firmly impacted in the right ventricular apex. The left ventricle was slightly hypertrophic, without cavity dilation (Figure 5), and small whitish vegetations could be seen on the free margin of the mitral valve leaflets. The coronary arteries showed no atherosclerotic lesions with significant luminal obstruction. The microscopic examination of the heart revealed fibrinous mitral valve vegetations with no inflammatory component, evidencing mild left ventricular myocardial sclerosis. The right renal parenchyma was extensively replaced by a large multilobular tumor formation, measuring 12 cm in its larger axis, consisting of whitish and firm tissue, with invasion to the perirenal fat, pelvis and renal vein (Figure 6). The microscopic examination evidenced carcinoma with intense cellular anaplasia, areas of necrosis and hemorrhage, in addition to vascular invasion (Figure 7). Both adrenal glands showed large metastases, measuring 8 cm and 7 cm, in the right and left glands, respectively, with complete replacement of the glandular parenchyma (Figure 8). In addition, multiple metastatic nodules were seen in the left kidney parenchyma and both lungs. The surface of the left kidney was finely granular and the microscopic examination evidenced benign nephrosclerosis and hyaline arteriolosclerosis. Other postmortem examination findings were: parathyroid adenoma, measuring 2.5 cm; pulmonary emphysema and chronic bronchitis; nodular prostatic hyperplasia; previous partial gastrectomy; small isolated focus of bronchopneumonia; and moderate aortic atherosclerosis. (**Luiz Alberto Benvenuti, MD**)

**Anatomopathological diagnoses:** Renal cell carcinoma of the right kidney, with multiple metastases (carcinomatosis); nonbacterial thrombotic endocarditis of the mitral valve; hypertensive heart disease; parathyroid adenoma; chronic obstructive pulmonary disease, with chronic emphysema and bronchitis; nodular prostatic hyperplasia. (**Luiz Alberto Benvenuti, MD**)

## Comments

The patient was a 76-year-old male smoker, who had systemic arterial hypertension, and was followed up since the age of 50 years. He developed heart failure and arrhythmias, being treated with drugs and ICD implantation in 2009 to prevent sudden death. At that time, imaging tests showed a solid nodule in the lower pole of the right kidney, measuring 2.9 cm. The patient was instructed to see an urologist to investigate that lesion. After three years, he was admitted to the emergency unit with consciousness lowering and hemodynamic shock of unknown etiology, and eventually died three days later.

The postmortem examination confirmed the presence of hypertensives heart disease, which seemed compensated, with neither left ventricular dilation nor acute pulmonary edema. In addition, there was neither ischemic heart disease nor any evidence of acute myocardial infarction. Thus, we do not believe the last clinical findings, which made him seek the emergency unit and eventually culminated in his death, could be attributed to cardiac causes.

In addition, disseminated malignant neoplasm originating from right kidney carcinoma and whose characteristics were compatible with renal cell carcinoma was detected. That type of tumor is usually extremely aggressive and has few symptoms.^17^ It is worth noting that, when that tumor was identified for the first time in 2009 and had no confirmed diagnosis, it was small and its resection could have been performed, leading eventually to the patient's cure. However, due to unknown reasons, the tumor was not investigated then. Renal cell carcinoma can originate metastases in several organs, such as the adrenal glands; however, the involvement of both glands is extremely rare.^18^ In the present case, the metastases were bilateral and extensive, completely replacing the glandular parenchyma. The microscopic examination showed not even a trace of the adrenal glands, and we can speculate that the patient's terminal clinical findings (consciousness lowering and hemodynamic shock) might relate to adrenal insufficiency.

It is worth noting that the postmortem examination evidenced nonbacterial thrombotic endocarditis of the mitral valve, whose etiology could have been the disseminated malignant neoplasm.^19^ (**Luiz Alberto Benvenuti, MD**)

**Section editor:** Alfredo José Mansur (ajmansur@incor.usp.br)

**Associated editors:** Desidério Favarato (dclfavarato@incor.usp.br)

Vera Demarchi Aiello (anpvera@incor.usp.br)
